# The fate of bone marrow-derived cells carrying a polycystic kidney disease mutation in the genetically normal kidney

**DOI:** 10.1186/1471-2369-13-91

**Published:** 2012-08-29

**Authors:** Elizabeth Verghese, Chad Johnson, John F Bertram, Sharon D Ricardo, James A Deane

**Affiliations:** 1 Biomedical and Health Sciences, Victoria University, St Albans, Australia; 2 Monash Immunology and Stem Cell Laboratories, Monash University, Clayton, Australia; 3 Monash Micro Imaging, Monash University, Clayton, Australia; 4 Department of Anatomy and Developmental Biology, Monash University, Clayton, Australia; 5 Current Address: The Ritchie Centre, Monash Institute of Medical Research, Monash University, Clayton, Australia

**Keywords:** Bone marrow-derived epithelial cells, Cyst, Hematopoietic stem cells, Polycystic kidney disease, Proliferation, Renal injury

## Abstract

**Background:**

Polycystic Kidney Disease (PKD) is a genetic condition in which dedifferentiated and highly proliferative epithelial cells form renal cysts and is frequently treated by renal transplantation. Studies have reported that bone marrow-derived cells give rise to renal epithelial cells, particularly following renal injury as often occurs during transplantation. This raises the possibility that bone marrow-derived cells from a PKD-afflicted recipient could populate a transplanted kidney and express a disease phenotype. However, for reasons that are not clear the reoccurrence of PKD has not been reported in a genetically normal renal graft. We used a mouse model to examine whether PKD mutant bone marrow-derived cells are capable of expressing a disease phenotype in the kidney.

**Methods:**

Wild type female mice were transplanted with bone marrow from male mice homozygous for a PKD-causing mutation and subjected to renal injury. Y chromosome positive, bone marrow-derived cells in the kidney were assessed for epithelial markers.

**Results:**

Mutant bone marrow-derived cells were present in the kidney. Some mutant cells were within the bounds of the tubule or duct, but none demonstrated convincing evidence of an epithelial phenotype.

**Conclusions:**

Bone marrow-derived cells appear incapable of giving rise to genuine epithelial cells and this is the most likely reason cysts do not reoccur in kidneys transplanted into PKD patients.

## Background

Polycystic kidney disease (PKD) is a common genetic condition (>1:1000 live births) involving the formation of renal cysts [1]. The kidney is a system of tubules and ducts formed by epithelial cells and it is these renal epithelial cells from which cysts originate in PKD. Increased proliferation of epithelial cells is a driver of cyst formation and a distinctive feature of PKD that is exemplified by the pathogenesis of the most common form of the disease, autosomal dominant PKD (ADPKD) [2,3]. In ADPKD, cysts have been reported to originate from the clonal expansion of epithelial cells with an inherited mutation plus an acquired somatic mutation of one of two ADPKD genes (*PKD1* or *PKD2)*[4-7]. Autosomal recessive PKD (ARPKD) is a rarer childhood onset form of the disease whose pathogenesis also involves high levels of epithelial proliferation [8].

Renal transplantation is one of the few viable treatment options for PKD patients. In light of continued, but somewhat controversial, reports that cells derived from hematopoietic stem cells in the bone marrow (BM) can integrate into the kidney and form epithelial cells [9-16], this transplant situation raises some interesting questions. A phenotypically normal donor kidney transplanted into a PKD afflicted recipient may incorporate epithelial cells originating from host BM cells carrying a PKD causing mutation. The mobilization of bone marrow hematopoietic stem cells and incorporation of BM-derived epithelial cells are also reported to be enhanced by renal injury which is a common occurrence during the transplantation process [9,11,12,17,18]. While there is a consensus that BM-derived cells are not major contributors to epithelial repair in the kidney, low level contributions have been reported in several recent studies [13-16]. The proliferative phenotype of PKD epithelial cells could also be expected to amplify the numbers of these cells after integration into the kidney. Despite these possibilities, the reoccurrence of cysts in a genetically normal donor kidney has not been reported in PKD patients. While obvious macroscopic cystic manifestations of engrafted PKD cells can presumably be excluded, the existence and phenotype of rare mutant epithelial cells in the tubule has not been specifically investigated. Previous studies of BM-derived renal epithelial cell have used genetically normal BM and relied on tracing the Y chromosome of male-derived BM cells in a kidney of female origin. The expression of epithelial markers by BM-derived cells has been used to infer an epithelial phenotype, although unambiguously determining whether a nuclear Y-chromosome signal and a cytoplasmic epithelial marker are in the same cell has proved difficult [19,20]. The expression of a cystic phenotype by BM-derived cells carrying a PKD mutation, even at a microscopic level, would aid detection and provide support for a genuine epithelial contribution. A lack of cystic phenotype expression by mutant BM-derived cell could either be interpreted as evidence that the PKD phenotype of mutant cells is being suppressed in the environment of the genetically normal kidney, or be due to the lack of a BM-derived contribution to renal epithelia. Distinguishing between these latter two outcomes requires an understanding of the phenotype (epithelial or otherwise) of BM-derived cells in the tubule. While BM-derived cells with a PKD mutation appear to be incapable of causing clinically relevant changes to the kidney, their phenotype is potentially of interest with regard to mechanisms of cystogenesis and sources of material for cell-based reparative strategies. We investigated the phenotype of BM-derived PKD mutant cells in the kidney using a controlled mouse model where tissue preparation and detection techniques could be optimized. Our objective was to investigate the nature of BM-derived cells in the kidney by testing whether mutant bone marrow-derived cells can express a cystic epithelial phenotype.

## Methods

### Experimental design

Wild type female mice underwent BM ablation and were transplanted with mutant BM from male mice that were homozygous for the PKD-causing Oak Ridge Polycystic kidney disease gene [21,22]. Recipient mice then underwent renal ischemia-reperfusion (IR) injury to induce transplanted BM cells to home to the kidney and give rise to renal epithelial cells. In this model, even a small contribution of BM-derived renal epithelial cells could potentially result in cystogenesis due to the proliferative advantage bestowed by the PKD-causing genetic defect. We assessed whether transplanted BM cells gave rise to renal epithelium and cysts using the male-specific Y chromosome to trace mutant BM-derived cells in conjunction with markers of renal epithelial phenotype.

### Mouse PKD model

All experiments were conducted under the Australian code of practice for the care and use of animals for scientific purposes and were approved in advance by a Monash University Animal Ethics Committee. Mice used were the Oak Ridge Polycystic Kidney Disease strain (*orpk*) on a C3H background, a recessive model of PKD. The genotype of mice was determined by PCR amplification of fragments of the mutant Tg737 or wild type gene from tail DNA [23].

### Sex mismatched bone marrow transplantation

Wild type female C3H mice (8–10 weeks) were irradiated with a 10 Gray dose of gamma radiation delivered as two 5 Gray doses separated by three hours, to ablate BM. After irradiation, recipient mice received 10^7^ whole BM cells from mutant male *orpk* donor mice with PKD (12–15 weeks) via the tail vein. We have previously used this protocol with GFP positive bone marrow and observed approximately 80% donor engraftment as measured in peripheral blood [24]. BM engraftment was verified by amplifying the male-specific *Sry* gene from the peripheral blood of recipients using the primers 5’-CAGCTAACACTGATCTTTC-3’ and 5’-TTACTGGCCAGAAT-3’ [9]. Body weight was also monitored as a measure of health.

### Induction of renal ischemia-reperfusion injury

Six weeks after BM transplantation unilateral renal ischemia was induced under isoflurane anesthetic as previously described [25]. Briefly, the left kidney was accessed by a flank incision and the renal pedicel clamped for 45 min using a specially designed vessel clip and forceps (S&T, Fine Science Tools, Switzerland). The clip was removed and reperfusion confirmed by a change in the color of the kidney from purple to red. The incision was sutured and anesthetic removed for recovery.

### Histology and Y chromosome FISH

Mice were perfusion fixed with 4% paraformaldehyde under anesthetic and kidneys collected 2 weeks (n = 2), 4 weeks (n = 3) or 12 weeks (n = 2) after the induction of injury. Kidneys were embedded in paraffin and 6 μm sections cut. Sections were dewaxed in xylene, rehydrated through graded alcohols to water and either stained with Periodic Acid Schiffs for histology, or processed for Y chromosome fluorescence in situ hybridization (FISH). For Y chromosome FISH, sections were incubated in 1 M sodium thiocyanate solution for 10 min at 80°C. They were washed in PBS and digested in 0.4% w/v Pepsin in 0.1 M HCl solution for 10 min at 37°C. The reaction was quenched in 0.2% glycine in 2X PBS solution. The sections were washed in PBS, post-fixed in 4% paraformaldehyde solution for 2 min, washed in PBS, dehydrated through a graded alcohol series and air dried. A TRITC labeled Y chromosome paint (Star-FISH, Cambio, Cambridge, UK Cat No 1200-YM Cy3-01) was added to each section, which were then cover-slipped and sealed with rubber cement. Slides were denatured at 65°C for 10 min and incubated overnight at 37°C in a humidified chamber. Slides were washed at 37°C in 3 changes of 50% formamide/ 2 X SSC and 2 X SSC for 5 minutes and 4 X SSC/ 0.05% Tween-20 for 10 minutes. Slides were then washed in PBS, incubated with either FITC labeled *Lotus tetragonolobus* agglutinin (Vector Laboratories, UK, Cat No.FL-1321) in PBS (1:50) for 2 hours at room temperature or underwent immunofluorescence staining for pan cytokeratin using a primary pan cytokeratin antibody (Dako Cytomation, Denmark) and a secondary Alexa fluor 647 labeled anti-rabbit antibody (Invitrogen, Carlsbad, CA).

### Quantification of Y chromosome-positive PKD mutant cells in the kidney

Co-localization of the Y chromosome FISH signal and DAPI staining in nuclei was used to identify BM-derived PKD cells in recipient kidneys. A Provis Fluorescence microscope (Olympus, Tokyo, Japan) was used to capture randomly selected fields (40 × objective, 231 × 173 μm) from the cortex and medulla of recipient kidneys 2 weeks, 4 weeks and 12 weeks post IR (2–3 mice per time point). The percentage of Y chromosome positive cells was calculated based on at least 500 nuclei for each time point.

### Analysis of co-staining for the Y chromosome and epithelial markers

High power images (100 × objective 92 × 69 μm or 40 × objective 231 × 173 μm) of male control and female experimental kidneys (2 weeks, 4 weeks and 12 weeks post IR) stained with Y chromosome FISH, epithelial markers and DAPI were captured on a Provis Fluorescence microscope (Olympus, Tokyo, Japan). Composite images were created using AnalySIS version 5.0 software (Olympus) to collate separate channels where the TRITC Y-chromosome signal was false colored green, the FITC conjugated LTA signal was false colored red and DAPI colored blue. Images were adjusted in a linear manner (brightness and contrast) if required.

To allow three dimensional reconstruction of the renal tubule, sequential confocal planes were imaged for each field using an Olympus FluoView 1000 confocal microscope (Olympus, Centre Valley, Pennsylvania). To create composite images TRITC Y-chromosome signals were false colored green, FITC and Alexa 647 were false colored red and DAPI colored blue. Images were adjusted in a linear manner (brightness and contrast) and three dimensional models of labeled cellular structures created using the “surface” function of IMARIS (Bitplane, AG, Zurich, Switzerland) at Monash MicroImaging. Images were compiled using Photoshop 5.5 (Adobe Systems, San Jose, CA).

## Results

### Confirmation of mutant PKD bone marrow engraftment in recipients

**Figure 1:**
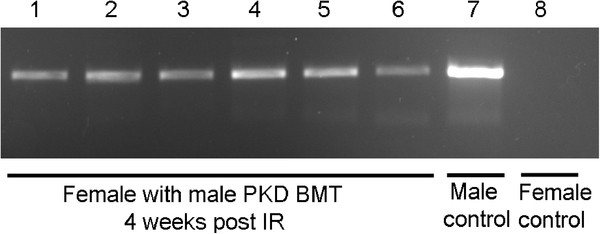
**PCR for the *Sry *gene confirms the engraftment of mutant bone marrow-derived cells in wild type mice.** PCR-based detection of the male-specific *Sry *gene in peripheral blood confirmed the engraftment of male bone marrow cells in female mice 4 weeks after transplantation. An 800 base pair band is present in all genetically normal females transplanted with male mutant bone marrow (Lanes1-6) and a male positive control (Lane 7), but is absent from an untransplanted female negative control (Lane 8).

Polymerase chain reaction (PCR)-based detection of the male-specific *Sry* gene in peripheral blood confirmed the engraftment of PKD mutant male BM in wild type female mice 4 weeks after transplantation (Figure 1). This result demonstrated that hematopoietic stem cells in the transplanted BM had repopulated the BM niche of recipients and were producing circulating blood cells. Similar results were observed 12 weeks after transplantation (data not shown). Confirming the specificity of this assay, the *Sry* gene was only detected in male positive control samples and was not present in female negative control samples. Mice also maintained body weight, another indicator of successful engraftment (data not shown).

### Wild type recipients of mutant bone marrow do not exhibit the renal pathology of PKD

**Figure 2:**
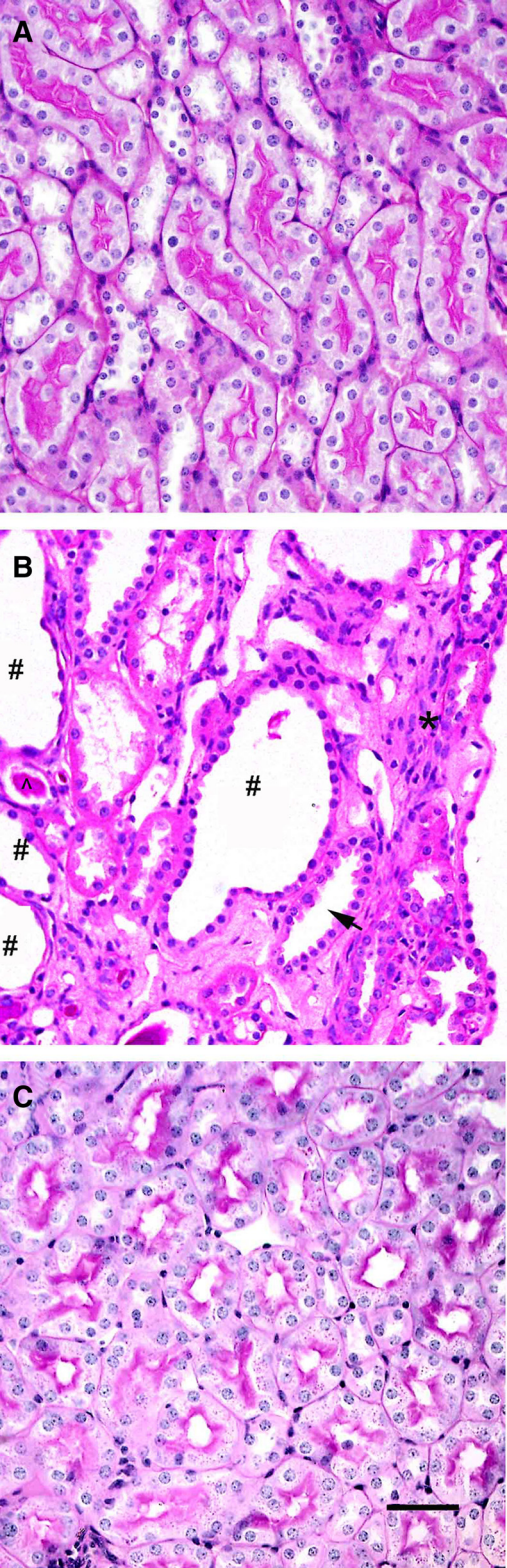
**Wild type recipients of mutant bone marrow do not exhibit PKD. (A** and **B**) Compared to an age matched wild type control (**A**), 12 wk old homozygous orpk mice (**B**) exhibited features of PKD including cysts (#), dilated tubules (arrow) and increase in the cellularity of the interstitium (*). (**C**) Wild type female mice transplanted with mutant bone marrow demonstrated complete renal repair 12 wks after injury and did not exhibit the histopathology associated with PKD. Scale bar in C = 100 μm and A & B are at the same magnification as C.

Compared to uninjured control kidneys (Figure 2A), kidneys of 12 wk old homozygous orpk mice used as BM donors exhibited signs of PKD such as cysts, dilated tubules and extracellular matrix expansion (Figure 2). Twelve weeks after renal IR, the kidneys of wild type recipients of mutant BM (Figure 2C) were comparable to uninjured control kidneys (Figure 2A). This suggests extensive renal repair and an absence of any major chronic pathology related to injury. No gross cystic changes consistent with the development of PKD were observed in the kidneys of wild type mice transplanted with mutant bone marrow and subjected to renal IR (Figure 2C).

### PKD mutant BM-derived cells are present in recipient kidneys

**Figure 3:**
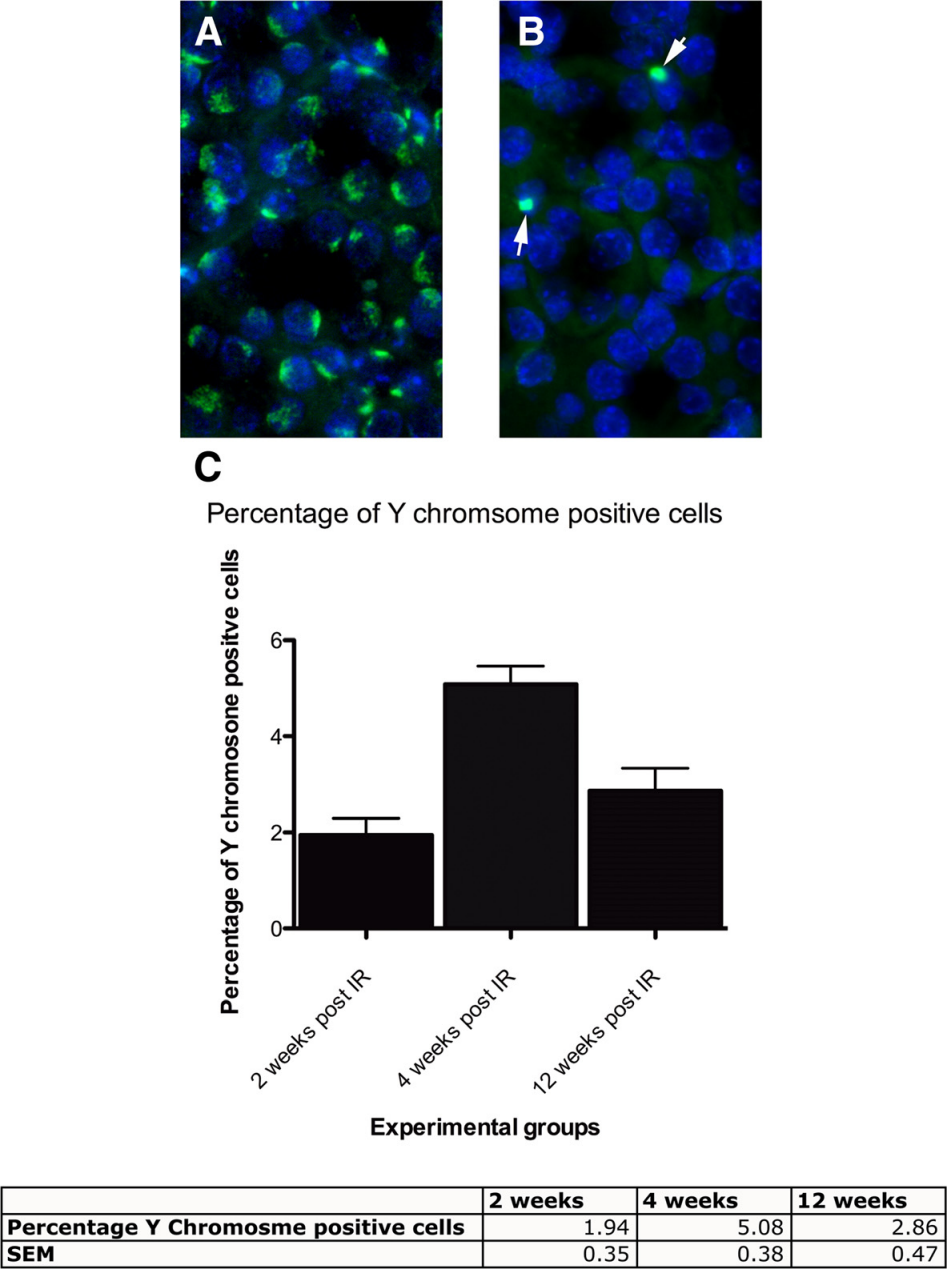
**Mutant Y chromosome positive cells derived from bone marrow are present in recipient kidneys.** (**A** and **B**) Representative fluorescence micrographs of Y chromosome-positive cells in the kidney are shown for a wild type male control (**A**) and a female recipient of male mutant PKD BM 4 weeks post IR (**B**). Nuclei are counterstained with DAPI (blue). (**C**) Quantification of Y chromosome-positive cells per field in transplanted females showed that BM-derived cells with the PKD mutation were detectable 2, 4 and 12 weeks post IR. Percentages of Y chromosome positive cells are shown as mean ± SEM for at least 10 fields and a total of 500 nuclei per time point. Sections assessed were from 2 mice at 2 weeks, 3 mice at 4 weeks and 2 mice at 12 weeks.

Y chromosome FISH detected a signal in the nuclei of most cells from male control kidney (Figure 3A). Detection rates were not 100%, even in male control kidney, because the Y chromosome is not always present in the remaining portion of sectioned nuclei. The presence of the Y chromosome in the kidneys of transplant recipients demonstrated that cells derived from transplanted mutant male BM were present in kidneys of genetically normal female transplant recipients (Figure 3B). Quantification showed that BM-derived cells carrying the Y chromosome in their nucleus were readily observable 2, 4 and 12 weeks after IR (Figure 3C).

### Co-staining for BM-derived cells and the proximal tubule epithelial marker LTA

**Figure 4:**
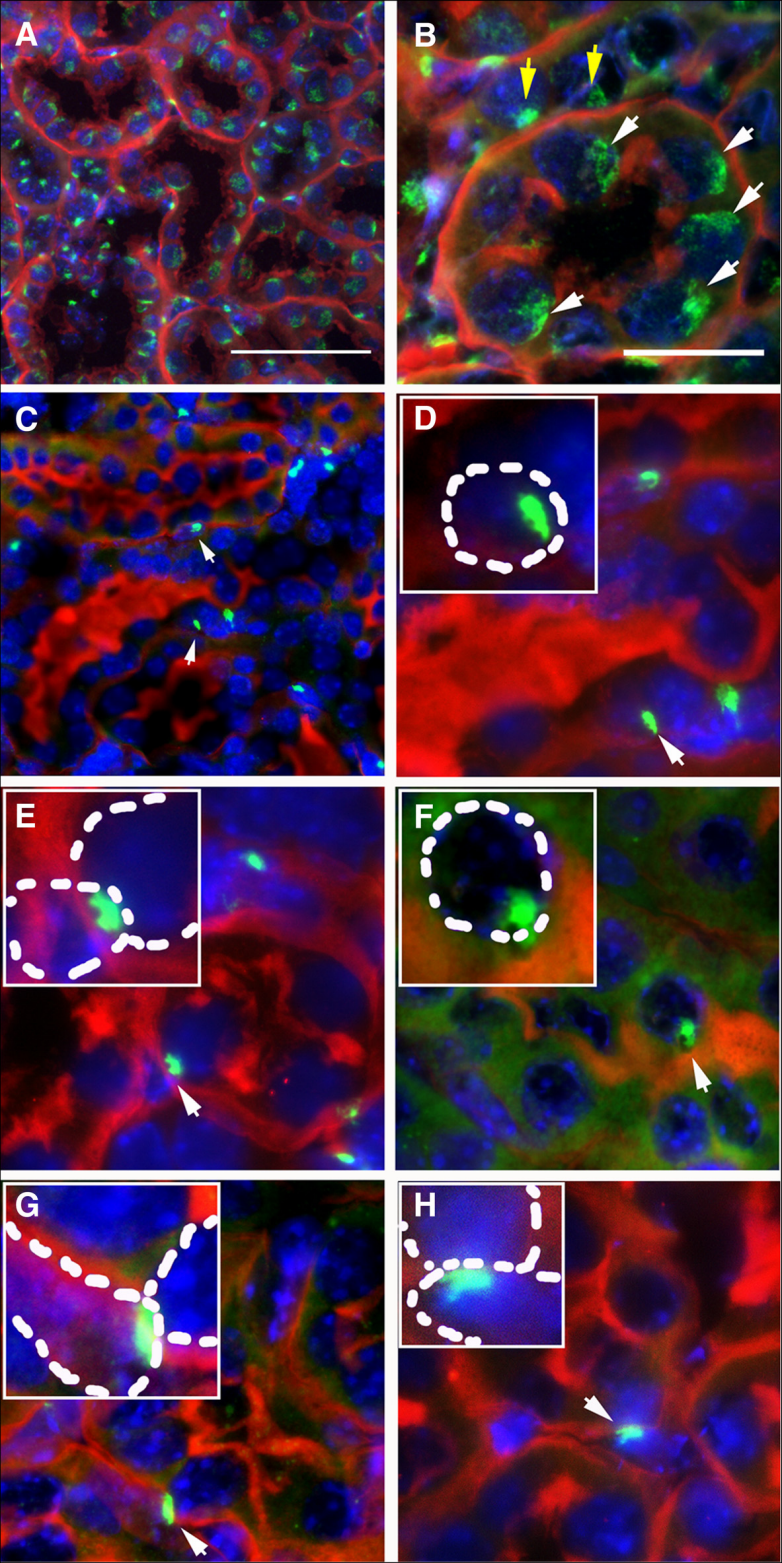
**Y chromosome FISH detection of mutant BM-derived cells and the epithelial marker LTA in the kidney.** (**A-H**) Conventional fluorescence micrographs show bone marrow-derived, PKD mutant cells as detected by the presence of a Y chromosome (green). Sections are costained with LTA as marker for the proximal tubule (red) and DAPI for nuclei (blue). (**A** and **B**) Male control kidney shows a Y chromosome signal in most cells (**A**) which is diffuse in the tubules (**B**, white arrows) and more compact in interstitial cells (**B**, yellow arrows). (**C**) A low power field from the kidney of a female transplanted with mutant male bone marrow showing several Y chromosome positive cells, some of which are associated with the tubule (arrows). (**D**-**H**) Examples of Y chromosome positive cells that could be interpreted as proximal tubule epithelial cells, but could equally be attributed to Y chromosome signals in the nuclei of infiltrating and overlaying non-epithelial cells as depicted by dashed lines in inserts. Scale bar in A = 50μm; C is at the same magnification as A. Scale bar in B = 20 μm; D-H are at the same magnification as B. Insets are at an additional 2X zoom.

The phenotype of BM-derived cells was further characterized by co-staining with the proximal tubule (the injury-susceptible initial segment of the renal tubule) epithelial marker *Lotus tetragonolobus* agglutinin (LTA) and fluorescence microscopy (Figure 4). Following treatment of sections for Y chromosome FISH, LTA gave linear staining of the outer extent of the proximal tubule and of the bush border on the luminal side of the tubule. In control male kidney, most LTA positive cells also contained a diffuse Y-chromosome signal, while interstitial cells between LTA positive epithelial cells contained a more compact Y chromosome signal (Figure 4A & B). In wild type female mice transplanted with mutant male BM, examples of BM-derived cells that were clearly interstitial were detected and displayed a compact Y chromosome signal as seen for interstitial cells in male control tissue (Figure 4C). Rarer examples of BM-derived cells were closely associated with the renal tubule, but their Y chromosome signal was compact as previously seen for interstitial cells (Figure 4D-H). The phenotype and precise location of these cells, as judged by LTA staining, was difficult to assess using conventional fluorescence microscopy (Figure 4D-H). From 120 examples of Y chromosome positive cells examined, only 10 cells were sufficiently entwined with the tubule that an epithelial phenotype might be considered (2 cells at 2 weeks, 5 cells at 4 weeks and 3 cells at 12 weeks). These frequencies and total numbers of Y chromosome-positive cells observed are consistent with previous reports of BM-derived cells in the tubule [13]. BM-derived cells were found as scattered examples in the renal tubule and did not appear as distinct clusters that might suggest a clonal origin and a proliferative phenotype.

### Three-dimensional reconstruction of BM-derived cells in the recipient kidney

**Figure 5:**
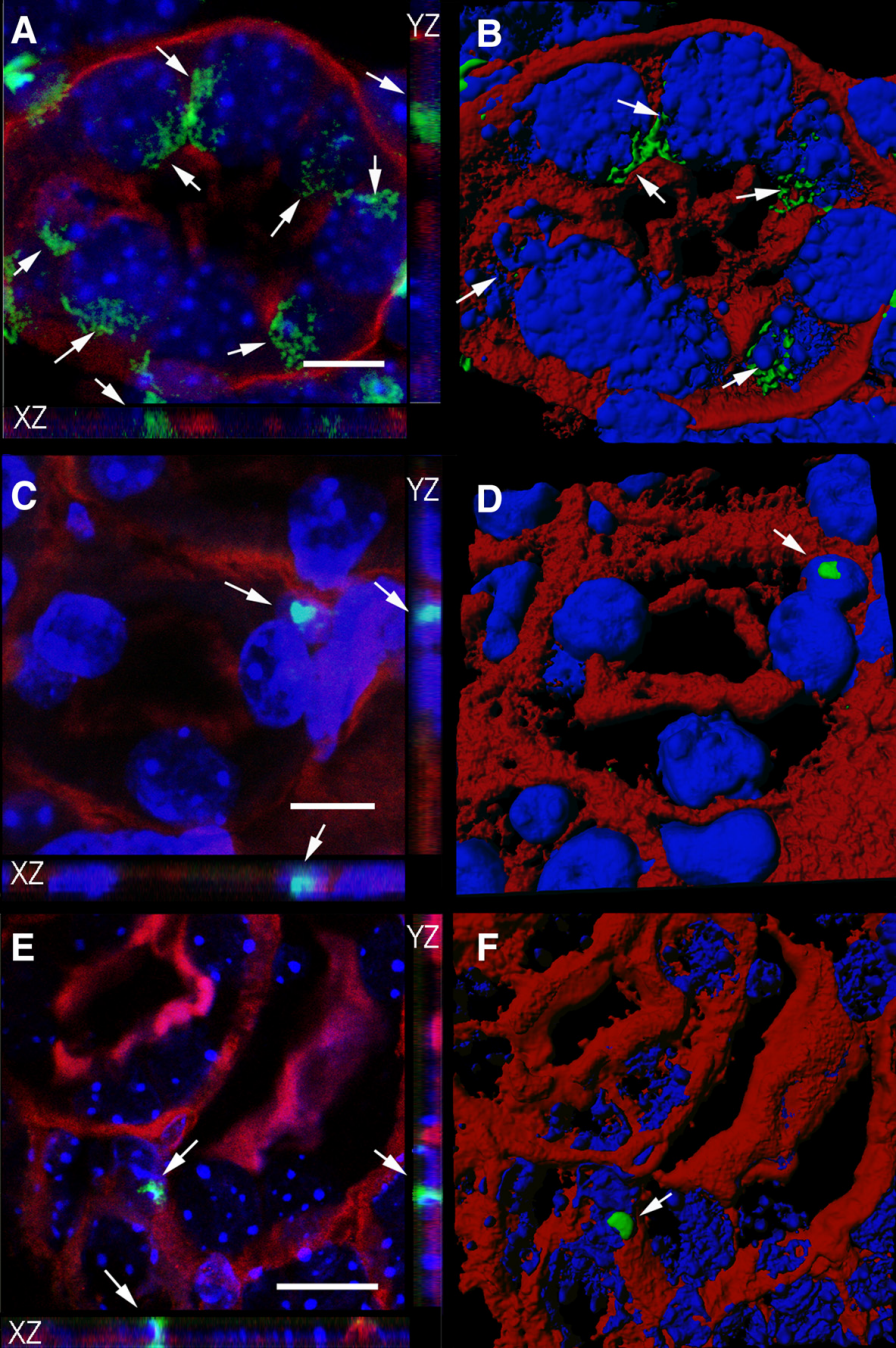
**Mutant bone marrow-derived cells in the recipient kidney do not express the proximal tubule epithelial marker LTA.** (**A-F**) FISH, confocal microscopy and three dimensional modeling was used determine the nature of Y chromosome staining (green) relative to the proximal tubule marker LTA (red) and DAPI stained nuclei (blue). Panels on the left (**A**, **C** and **E**) are confocal images with XZ and YZ planes shown as side panels. The corresponding panels on the right (**B**, **D** and **F**) are 3D reconstructions of these confocal images. (**A** and **B**) A cross-section of a tubule in a control male kidney showing dispersed Y chromosome signals in cells with typical epithelial morphology and LTA staining (arrows). (**C-F**) Y chromosome positive mutant cells in wild type female recipient kidney. (**C** and **D**) An example of a Y chromosome-positive cell (arrows) within the bounds of the proximal tubule that is not the same size or morphology as surrounding host tubular epithelial cells and contains a more compact Y chromosome signal. (**E** and **F**) A cell with a compact Y chromosome signal closely bordering the proximal tubule, but failing to express LTA. C and D are 4 wks after IR and E and F are 2 weeks after IR. Scale bars = 10 microns.

**Figure 6:**
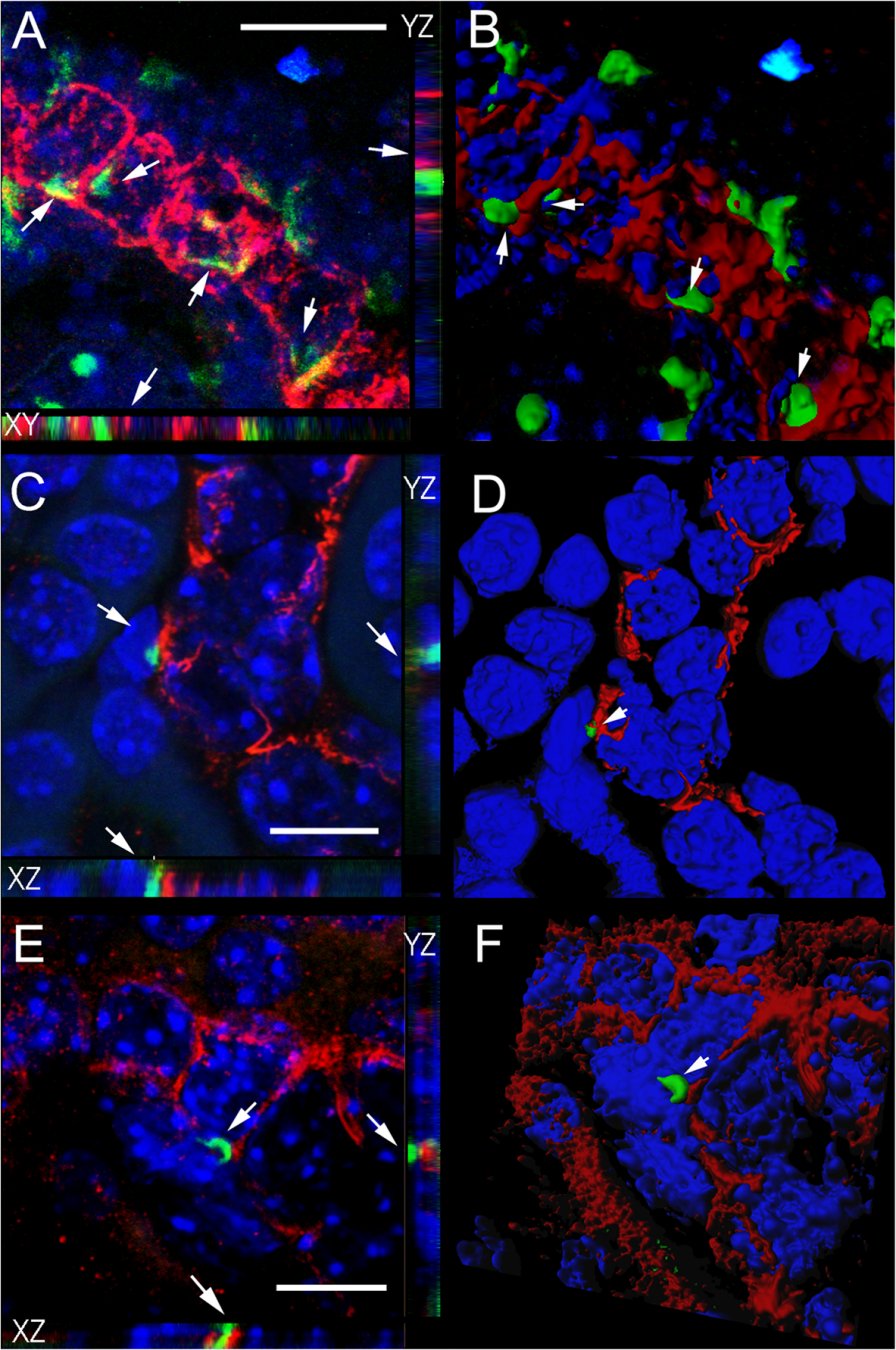
**Mutant bone marrow-derived cells in the recipient kidney do not express the epithelial marker pan cytokeratin.** (**A-F**) FISH, confocal microscopy and three dimensional modeling was used determine the nature of Y chromosome staining (green) relative to the epithelial marker pan cytokeratin (red) and DAPI stained nuclei (blue). Panels on the left (**A**, **C** and **E**) are confocal images with XZ and YZ planes shown as side panels. The corresponding panels on the right (**B**, **D** and **F**) are 3D reconstructions of these confocal images. (**A** and **B**) A longitudinal-section of a tubule in a control male kidney showing dispersed Y chromosome signals (arrows) in cells with typical epithelial morphology and pan cytokeratin staining. (**C-F**) Y chromosome-positive mutant cells from the bone marrow (arrows) were found in close proximity to recipient pan cytokeratin positive epithelial cells, but failed to stain for this marker themselves. C and D are 4 wks after IR and E and F are 12 weeks after IR. Scale bars = 10 microns.

Confocal microscopy and three-dimensional reconstruction allowed detailed examination of the colocalization of BM-derived cells with the proximal tubule marker LTA (Figure 5), or the more general epithelial marker pan cytokeratin (Figure 6). Male positive control mice clearly showed Y chromosome positive epithelial cells which expressed LTA (Figure 5A & B) or pan cytokeratin (Figures 6A & B). Y chromosome positive cells that did not express epithelial markers were also visible in the interstitial region between tubules (Figures 5A &6A). As previously seen by conventional fluorescence microscopy, the Y chromosome signal of interstitial cells was compact as opposed to the more diffuse signal seen in epithelial cells. There were some examples of Y chromosome positive BM-derived cells within the bounds of the renal tubule, but these cells were not the same size or morphology as renal tubular cells and contained a Y chromosome signal that was compact like that of male interstitial cells (Figure 5C & D). There were also examples of Y chromosome positive cells closely associated with the renal epithelial layer. Careful examination of these cells did not provide convincing evidence of the expression the renal epithelial markers LTA (Figure 5C-F) or pan cytokeratin (Figure 6C-F) and suggested that these cells were not epithelial. Once again, the Y chromosome signal from these cells was compact like that of interstitial cells. No convincing examples of BM-derived renal epithelial cells were detected despite the detailed examination of 14 candidate Y chromosome positive cells from the kidney 2 weeks, 4 weeks and 12 weeks after IR. The numbers of cells we considered (but ultimately dismissed) as possible BM-derived epithelial cells is comparable to the number of BM-derived epithelial cells previously reported in a similar model not involving mutant BM cells [13].

## Discussion

We investigated the fate of mutant BM-derived cells with the potential to express a PKD phenotype in the genetically normal kidney. Previous studies suggest that renal injury is required to induce BM-derived cells to home to the kidney and give rise to renal epithelium [10,12]. The duration of renal ischemia and post ischemic recovery time have been reported to affect BM engraftment [18]. In the current study, recipient females underwent ischemia for 45 min, a length of time that causes substantial but repairable damage that has previously been reported to promote engraftment [9,12]. A number of post-ischemic recovery times were used to provide a wide window of opportunity to detect BM-derived renal epithelium and any resulting cystogenesis. The shorter post ischemic time points studied (two and four weeks) assessed whether a short proliferative burst due to ischemic injury might facilitate transient engraftment [26]. The longer time point (twelve weeks) assessed whether an extended post-ischemic recovery time increased the window for engraftment and allowed more time for cystic pathology to develop [18]. Thus our experiments were designed to maximize the chances of observing BM-derived renal epithelial cells in the recipient kidney.

Despite successful BM-engraftment and recovery from renal injury, histological examination did not detect any polycystic changes in the kidneys of wild type recipients of mutant bone marrow. In the absence of any gross pathology indicating cystogenesis, we used FISH to locate Y chromosome positive, BM-derived cells in the kidney. BM-derived cells were consistently found in the kidney 2, 4 and 12 weeks after injury. The BM-derived cells we detected failed to exhibit the proliferative phenotype that is characteristic of epithelial cells in PKD.

Since BM-derived cells failed to express a cystic features that would indicate an epithelial phenotype, we were limited to using the previously published combination of Y-chromosome FISH combined with epithelial markers to determine their nature. Conventional fluorescence microscopy of Y chromosome FISH combined with the epithelial marker LTA detected BM-derived cells that could be interpreted as being epithelial cells in the tubule. These cells were found at a low frequency that is consistent with previous reports of BM-derived epithelial cells [13]. However the compact Y chromosome signal in these BM-derived cells was as seen for interstitial cells. To our knowledge, this qualitative difference in the Y-chromosome signal obtained from epithelial versus interstitial cells on the kidney has not previously been used to aid in determining the phenotype of BM-derived cells. Confocal microscopy with three dimensional reconstruction also showed BM-derived cells within the boundary of, or closely appressed to, renal tubules. However, closer examination showed that in addition to having a compact Y chromosome signal, these cells differed from host renal epithelial cells with respect to their morphology and lack of clear epithelial marker expression. This is in contrast to epithelial cells in PKD which continue to express the epithelial markers of the nephron segment from which they originate [27]. Collectively these results suggest that, despite our efforts to provide conditions favoring their formation and detection; in the situation we studied BM-derived cells did not give rise to renal epithelial cells. We did not further investigate the nature of BM-derived cells detected, but based on previous studies they are likely to be macrophages, endothelial cells or myofibroblasts [24,28,29].

Despite reports of BM-derived renal epithelial cells in humans and animal models, there are also studies suggesting that these cells do not exist. Indeed, the apparent absence of BM-derived renal epithelial cells in our study is consistent with previous reports where the fate of non-PKD BM-derived cells has been traced in the kidney [13,19]. While the rarity of BM-derived epithelial cells could conceivably hamper detection, our experimental system is arguably more sensitive due to the potential of mutant BM-derived cells to express a proliferative phenotype should they give rise to epithelial cells. Many of the initial studies in this area appear to have concluded that there is a BM-derived contribution to renal epithelia due to unreliable tracing and phenotype determination techniques [19]. We have also noted a qualitative difference in the pattern of the Y chromosome signal in tubular versus interstitial cells that aids determination of phenotype. The examples of BM-derived cells in the kidney that we have presented here could be misinterpreted as having an epithelial phenotype if not carefully examined. Even if more definitive evidence of BM-derived cells with epithelial characteristics was present, the possibility remains that these cells are the product of fusion between a BM-derived cell and a renal epithelial cell [30]. While we did not detect cells fitting this description in our experiments, fusion with genetically normal epithelial cells would be likely to rescue the proliferative phenotype carried by mutant BM and reduce the probability of detection.

## Conclusions

The results from our mouse model add weight to suggestions that cells derived from hematopoietic stem cells in the bone marrow cannot cross lineage barriers to form renal epithelia. Extending this result to human renal transplantation, we suggest that the most likely explanation for the failure of PKD to reoccur in nonpolycystic renal grafts is due to the absence of a genuine epithelial contribution to the transplanted kidney from the host bone marrow. The apparent inability of hematopoietic stem cells to give rise to renal epithelia is disappointing given the search for convenient sources of stem cells for renal reparative therapies [31]. The kidney’s major capacity for epithelial repair appears to rely on the proliferation of intrinsic renal epithelial cells [19,32]. The most likely contribution of BM-derived cells, such as macrophages and endothelial progenitors, is to provide a supportive environment for endogenous epithelial re-establishment [24,29,33].

## Competing interests

The authors declare that they have no competing interests.

## Authors’ contributions

JAD, EV, JFB and SDR designed the experiments. EV and JAD performed the experiments. CJ assisted EV with confocal imaging and with preparing 3D reconstructions. JAD and EV prepared the manuscript with advice from SDR and JFB. All authors read and approved the final manuscript.

## Pre-publication history

The pre-publication history for this paper can be accessed here:

http://www.biomedcentral.com/1471-2369/13/91/prepub
